# Sequential Release HydroLipo System for STING Gene Epigenetic Reprogramming and Immune Activation in Glioblastoma

**DOI:** 10.1002/advs.202408323

**Published:** 2024-12-11

**Authors:** Hao Yu, Wenjing Liu, Kaikai Ding, Jiangjie Wu, Cheng Wang, Siyuan Wang, Lingyun Wu, Qiuying Tang, Xin Yin, Kan Jiang, Danfang Yan, Xu Wang, Si Chen, Senxiang Yan

**Affiliations:** ^1^ Department of Radiation Oncology the First Affiliated Hospital Zhejiang University School of Medicine 79 Qingchun Road Hangzhou Zhejiang 310003 P. R. China; ^2^ College of Materials Science and Engineering Zhejiang Key Laboratory of Plastic Modification and Processing Technology Zhejiang University of Technology Hangzhou 310014 P. R. China

**Keywords:** epigenetic reprogramming, glioblastoma, sequential release hydroLipo system, STING

## Abstract

Glioblastoma (GBM) remains a daunting oncological challenge because of its aggressive nature and resistance to conventional therapies. Inhibition of the intrinsic STING pathway in GBM hampers the effectiveness of immunotherapies. To overcome this clinical limitation, a Sequential Release HydroLipo System (SRHLS) is developed, in which hydrogels and nanoparticles are combined for controlled drug release. The SRHLS sequentially released decitabine and STING agonists, thereby correcting STING signaling dysfunction through epigenetic reprogramming and enhancing antitumor immunity. According to in vitro and in vivo experiments, the SRHLS reshaped the tumor microenvironment and markedly inhibited tumor growth, recurrence, and metastasis. These findings underscore the potential of the SRHLS as a promising therapeutic strategy for GBM.

## Graphical abstract

The Sequential Release HydroLipo System (SRHLS) offers a dual‐phase, delayed‐release approach, combining decitabine and STING agonists to address STING pathway deficiencies in glioblastoma. By optimizing drug delivery, this system minimizes side effects and costs while promoting sustained antitumor effects and remodeling the tumor microenvironment, providing a promising strategy to enhance immunotherapy outcomes in glioblastoma.

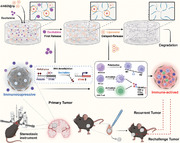

## 1 Introduction

Glioblastoma (GBM), the most prevalent and deadly primary brain cancer in adults, represents 50% of all gliomas.^[^ ^1^, ^2^, ^3^ ^]^ The standard treatment regimen for GBM encompasses surgical resection, followed by concurrent chemoradiotherapy and subsequent adjuvant chemotherapy. Temozolomide is the preferred chemotherapeutic agent for GBM. Its combination with radiotherapy significantly enhanced survival rates compared to radiotherapy alone, with a 2‐year survival rate of 27.2% versus 10.9%, and a 5‐year survival rate of 9.8% versus 1.9% (HR 0.6 [95% CI, 0.5–0.7]; P < 0.001).^[^ ^4^ ^]^ However, despite the availability of standard treatment, median survival after GBM diagnosis is only 14–16 months, with an even poorer prognosis for elderly patients, whose average survival is < 8.5 months.^[^ ^5^ ^]^ Given the poor prognosis, new therapeutic strategies need to be urgently developed for GBM patients.

Immunotherapy is a rapidly advancing field in cancer treatment. Unfortunately, GBM does not respond significantly to this approach. For instance, in phase III trials, antibodies targeting immune checkpoint inhibitors such as PD‐1 (e.g., nivolumab or pembrolizumab) caused no improvement in overall survival.^[^ ^6^, ^7^ ^]^ The highly immunosuppressive and non‐immunogenic tumor microenvironment (TME) of GBM is speculated to be a major factor underlying its resistance to immunotherapy. Transforming immunosuppressive “cold” tumors into immunologically active “hot” tumors for enhancing the immune response is a promising strategy. The STING pathway is gaining attention as a promising immunotherapy target due to its strong local immunostimulatory effects. However, functional mutations or epigenetic silencing of the STING gene promoter, such as promoter hypermethylation, often impair STING signaling in GBM cells, likely contributing to intrinsic immunosuppression.^[^ ^8^ ^]^

Decitabine (DAC), a DNA methyltransferase inhibitor (DNMTi).^[^ ^9^ ^]^ has exhibited the potential to restore functional STING signaling by inhibiting DNA methylation in at least half of the melanoma cell lines tested, augment immune recognition, and kill tumor cells by upregulating MHC I molecules.^[^ ^10^ ^]^ This results in improved STING activation and type I interferon induction by STING agonists, which leads to tumor regression in mouse models through CD8+ T cell‐dependent immune responses.^[^ ^11^ ^]^ Therefore, a system that first releases DAC to activate STING signaling, followed by a slow release of STING agonists to reverse immunosuppression, must be developed for effective GBM treatment. However, clinical challenges in achieving this goal include frequent injections, high drug doses, severe side effects, and additional economic costs.

**Scheme 1:**
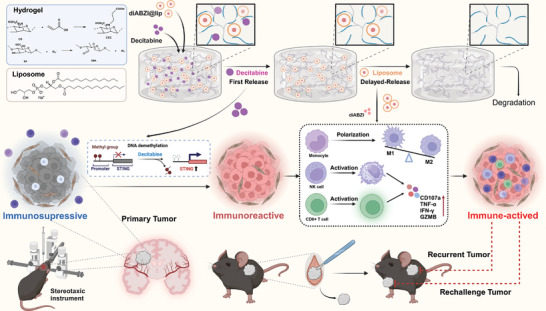
Schematic illustration of SRHLS‐induced antitumor therapy.

Hydrogels are 3D polymer networks with a high water retention capacity. They are ideal drug delivery systems because of their biocompatibility and tunable properties.^[^ ^12^ ^]^ However, when delivering multiple drugs, single hydrogel networks allow limited control over drug release rates and offer poor targeting capabilities.^[^ ^13^, ^14^ ^]^ To achieve controlled release times for different drugs and enhance therapeutic targeting, we combined hydrogels with nanoparticles.^[^ ^15^, ^16^ ^]^ In this approach, anionic liposomes were encapsulated within a cationic hydrogel to construct a sophisticated Sequential Release HydroLipo System (SRHLS). The SRHLS initially releases DAC completely, thereby enabling epigenetic reprogramming to correct STING signaling dysfunction in GBM cells, and followed by the gradual release of diABZI (a novel non‐nucleotide‐based ligand that potently activates STING) to induce a robust and sustained immune response. Single‐cell RNA sequencing (scRNA‐seq) and the in vivo orthotopic GBM model demonstrate that the SRHLS effectively augments the tumor‐killing function of NK and T cells and reprograms tumor‐associated macrophages (TAMs) to a tumor‐suppressive phenotype. This highlights the potential of the prepared SRHLS as an innovative approach to advancing GBM immunotherapy (**Scheme** **1**).

## 2 Results and Discussion

### 2.1 Fabrication and Characterization

**Figure 1:**
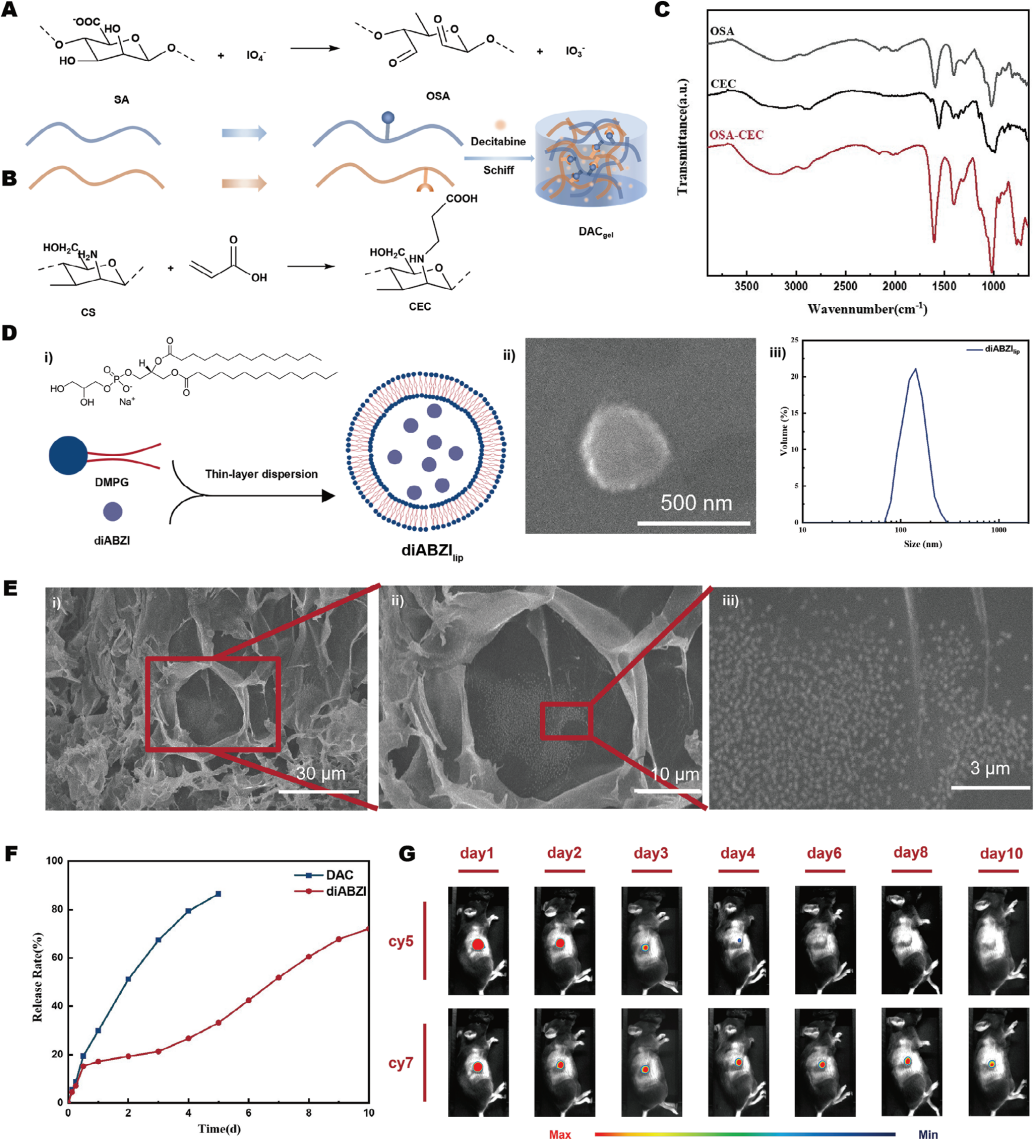
Preparation and characterization of SRHLS (A) the preparation of OSA. B) the preparation of CEC. C) FTIR spectra for the OSA, CEC, and OSA‐CEC hydrogels. D) the (i) preparation, (ii)SEN images, and (iii) size distribution of liposomes. E) SEM images of SRHLS from (i) the overall view and (ii and iii) the local view. F) Release curves of DAC and diABZI releasing from hydrogel and SRHLS. G) In vivo luciferase fluorescence imaging for the mice after SRHLS injection with progress of time.

The naturally derived biological macromolecule sodium alginate (SA) is typically used to fabricate hydrogel materials because of its superior biocompatibility, abundant carboxyl groups, and favorable solubility. Oxidized sodium alginate (OSA) obtained through SA oxidation includes aldehyde groups, which significantly enhance the capacity of SA to crosslink with other molecules (**Figure** **1**A).^[^ ^17^ ^]^ Chitosan is formed through partial deacetylation of the natural polysaccharide chitin. It is the only positively charged natural polysaccharide available and has the attributes of biodegradability, biocompatibility, and antibacterial activity.^[^ ^18^ ^]^ However, its application is limited by its reduced water solubility. The crystallinity of carboxyethyl chitosan (CEC) diminished when a specific quantity of hydrophilic carboxyl group was incorporated to augment the water solubility of chitosan (Figure 1B).

Concurrently, the remaining ‐NH2 in CEC can interact with ‐CHO of OSA via the Schiff base to rapidly form a hydrogel with a network structure. Following the uniform blending of 2 wt.% CEC and 10 wt.% OSA aqueous solution by using a double syringe in an equivalent volume, a CEC‐OSA hydrogel was swiftly achieved in ≈15 s. In Fourier transform infrared spectroscopy, carboxylate, the Schiff base, and amide peaks were superimposed at 1590 cm^−1^ to produce a hydrogel suitable for encapsulating drugs and liposomes (Figure 1C). 1,2‐Dimyristoyl‐SN‐3‐phospho‐rac‐(1‐glycerol) sodium salt (DMPG) was used to synthesize negatively charged hydrophilic cephalic liposomes. It can interact with chitosan, a cationic hydrogel substrate, forming a liposome reservoir through non‐covalent bonds within the hydrogel to circumvent premature drug release. scanning electron microscopy (SEM) images of the liposomes prepared through thin‐film hydration exhibited spherical multilayer vesicles that can encapsulate therapeutic drugs. Based on SEM results coupled with dynamic light scattering data, we found that the liposome diameter was 200 nm and the particle size was uniform (Figure 1D).

A specific quantity of liposome solution was blended into an OSA solution, and this mixture was then uniformly combined with a CEC solution using an equal‐ratio dual syringe system to create the SRHLS. In SEM images, the CEC‐OSA hydrogel matrix network exhibits a uniform distribution, with liposomes evenly dispersed throughout this hydrogel matrix (Figure 1E). The injectable gelation method not only ensures the convenience of drug delivery but also aids in achieving thorough mixing between liposomes and the hydrogel matrix due to the shear forces generated during the injection process. Additionally, the electrostatic interaction between negatively charged liposomes and positively charged hydrogel matrix further stabilizes the liposomes within the hydrogel, effectively preventing their aggregation, thereby forming a stable and uniform liposome reservoir within the hydrogel.

To further characterize the release efficacy of the SRHLS, the release environment was simulated in PBS solution in vitro for investigation. The release curve revealed that DAC was gradually released to a release rate of > 80% in the system during the initial 5 days. The release of diABZI is delineated into four stages. In the initial stage, non‐encapsulated drugs are promptly released into the environment. In the subsequent stage, the drug release rate is sluggish because of the anchoring effect of the liposome reservoir and chitosan substrate. In the third stage, the weakening of the anchoring effect occurs over time due to factors such as charge neutralization or structural changes in the liposomes, resulting in an accelerated release rate of diABZI into the environment. In the final stage, the release rate again decreases because the liposome reservoir is depleted. (Figure 1F). To verify the in vivo release efficiency of SRHLS, fluoresceins CY5 and CY7 were respectively loaded onto hydrogels and liposomes to simulate the release of two drugs. Continuous small animal imaging revealed that CY5 was nearly completely released within the first 5 days, whereas CY7 demonstrated sustained and stable release for at least 10 days (Figure 1G). These findings indicate that SRHLS effectively meets the therapeutic goal of sequentially releasing two drugs, ensuring the desired release kinetics for effective treatment.

### 2.2 Restoration of STING Gene Expression Through DNA Demethylation in GBM Cell Lines Enhances Antigenicity and Antitumor Immunity

**Figure 2:**
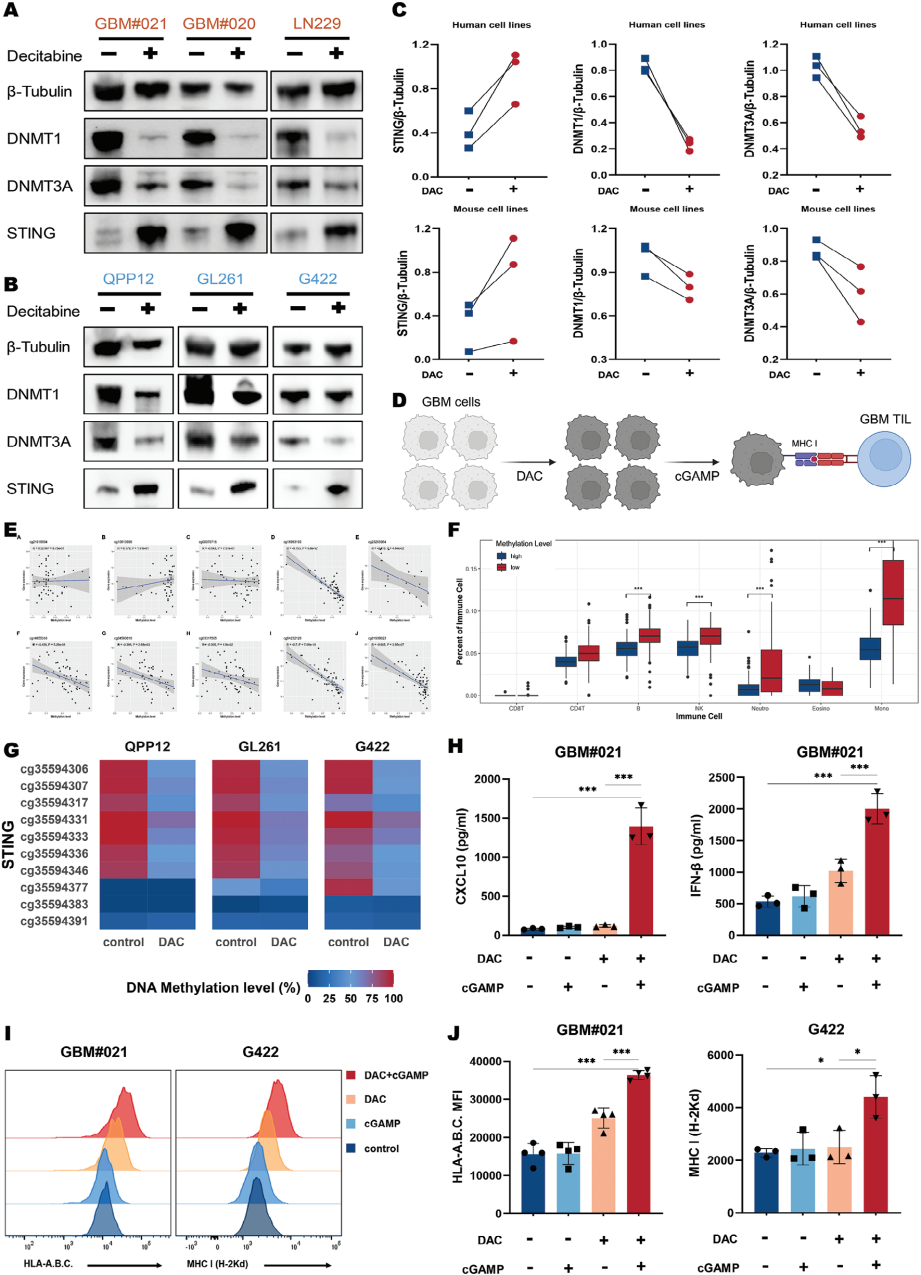
DNA demethylation restores STING expression in GBM cells, boosting antigenicity and antitumor immunity. A,B) Western blot analysis was conducted to evaluate DNMT and STING expression in lysates (15 µg) prepared from human and mouse cell lines, with or without DAC treatment. β‐Tubulin served as the loading control. C) The ratio of total STING and DNMT relative to β‐Tubulin was calculated for each cell line, with and without DAC treatment. D) A schematic illustrates tumor cell antigen presentation and immune cell activation via MHC‐I following DAC treatment (created with BioRender.com). E) Correlation between methylation levels at STING gene sites and STING gene expression. F) The inherent methylation of the STING gene influences antitumor immunity in GBM patients. G) Pyrosequencing analysis confirmed that hypermethylation of the STING gene in mouse GBM cell lines was reversed by DAC treatment. H) ELISA measurements of CXCL10 and IFN‐β concentrations were taken from supernatants of GBM#021 cells, with and without DAC treatment (*n* = 3). I,J) Representative histograms and mean fluorescence intensity (MFI) of HLA‐A.B.C (human) and MHC‐I (mouse) expression were analyzed in GBM cell lines (*n* = 4). p < 0.05 was considered statistically significant (^*^ *p* < 0.05, ^**^ *p* < 0.01, and ^***^ *p* < 0.001).

To evaluate whether promoter demethylation restores STING gene expression, six GBM cell lines (human cell lines: GBM#020, GBM#021, and LN229, and mouse cell lines: QPP12, GL261, and G422, with GBM#020, GBM#021, and QPP12 being the primary cell lines) were treated with DAC. According to immunoblot analysis, STING gene expression was downregulated or absent in the GBM cell lines, but all DAC‐treated cell lines exhibited varying degrees of STING expression reconstitution, along with a significant reduction in DNMT1 and DNMT3A expression (**Figure** **2**A–C). The relationship between methylation levels at various sites of the human STING gene and expression of this gene were analyzed using data from the EWAS Data Hub (https://ngdc.cncb.ac.cn/ewas/datahub/index). A significant negative correlation was observed at most sites, with the strongest correlation observed at the CpG site cg16983159 in the STING promoter (r = −0.753, P = 5.8e‐12) (Figure 2E).

Furthermore, we analyzed data from the GEO database dataset GSE143843 including 315 GBM patients. Based on the methylation levels at the cg16583159 site, the patients were segregated into high and low methylation groups. Analysis using the EpiDISH package in R revealed markedly differences in immune infiltration between two groups, showing that the high methylation group exhibited considerably lower levels of immune infiltration compared to the low methylation group. This indicated that the inherent methylation level of the STING gene in the tumor profoundly affects antitumor immunity (Figure 2F). Next, a DNA methylation profiling analysis of the STING gene in three mouse GBM cell lines was performed through pyrosequencing before and after DAC treatment to determine whether the mouse cell lines had the same characteristics as the human cell lines. STING promoter hypermethylation (as indicated by β values) was observed in the mouse cell lines, and methylation levels markedly decreased after DAC treatment (Figure 2G).

Next, we explored the immunostimulatory effects of a DAC‐based combination therapy with a STING agonist. CXCL10 has a potent chemotactic effect on activated lymphocytes, thereby playing a crucial role in regulating both innate and adaptive immunity.^[^ ^19^ ^]^ Interferon‐beta (IFN‐β) is a key type I interferon cytokine coordinating innate immune responses to infections, tumors, and inflammation.^[^ ^20^ ^]^ The induction of STING‐dependent CXCL10 and IFN‐β in cell culture supernatants was measured. The cell lines untreated with DAC showed no response to 2′3“‐cGAMP stimulation. However, following sequential DAC pretreatment and 2′3”‐cGAMP stimulation, CXCL10, and IFN‐β expression strongly increased, confirming STING signaling activation (Figure 2H).

Whether DAC‐mediated STING signaling in GBM cells could improve their antigenicity was investigated. A meaningful increase in human leukocyte antigen A, B, C (HLA‐A, B, C) was noted on the surface of human cell lines, and in MHC‐I molecules was observed on the surface of mouse cell lines during combination therapy (Figure 2I,J). This indicates that restoring STING gene expression is necessary for 2′3'‐cGAMP‐induced MHC‐I upregulation (Figure 2D). Given the significant role of HLA and MHC‐I in tumor antigen presentation,^[^ ^21^ ^]^ these findings suggest that epigenetic reprogramming of intrinsic STING signaling in GBM is a crucial player mediating effective antitumor immune responses.

### 2.3 Sequential Delivery of DAC and STING Agonist Through the SRHLS Synergistically Activates Immune Responses and Inhibits Tumor Progression In Vivo

**Figure 3:**
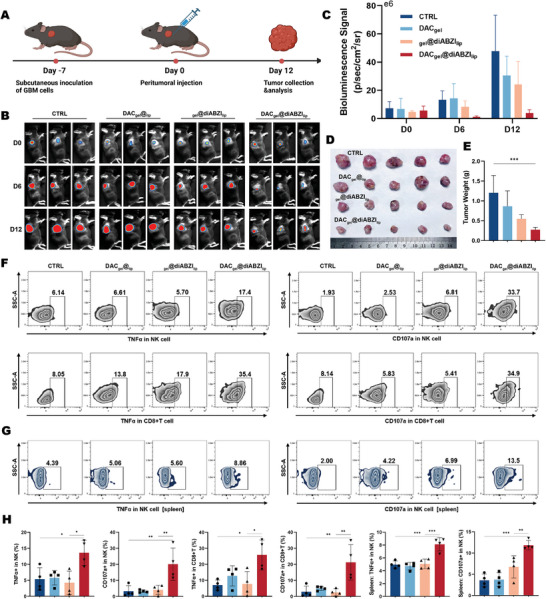
In vivo therapeutic effect of SRHLS in a subcutaneous GBM model in C57BL/6 mice. A) Schematic of the treatment protocol. B‐C) In vivo luciferase fluorescence imaging before and after treatment with DAC_gel_@_lip_, _gel_@diABZl_Iip_, and DAC_gel_@diABZI_lip_ (*n* = 3). D) Bright‐field images of excised tumor tissues (*n* = 5). E) Comparison of tumor tissue weights post‐excision (*n* = 5). F,G) TNF‐α and CD107a expression patterns in infiltrating NK and CD8+ T cells within tumors (F) and spleens (G), *n* = 4. H) Flow cytometry results analysis for each group (*n* = 4). ^*^ *p* < 0.05, ^**^ *p* < 0.01, ^***^ *p* < 0.001.

To investigate the therapeutic effects of sequential demethylation and STING agonist stimulation in vivo, a subcutaneous model was established by using luciferase‐labeled GBM cells in immunocompetent C57BL/6 mice. The tumors were treated with peritumoral SRHLS injections (**Figure** **3**A). According to imaging results, rapid tumor growth was noted in control mice. However, by the 6th day after treatment, the average fluorescence intensities of the DAC_gel_@_lip_, _gel_@diABZI_lip_, and DAC_gel_@diABZI_lip_ groups were 107.3%, 63.0%, and 9.2% of the control group, respectively, with P‐values of 0.88, 0.24, and 0.02. By the 12th day, the DAC_gel_@diABZI_lip_ group displayed considerably greater tumor inhibition, with fluorescence intensities of the control, DAC_gel_@_lip_, and _gel_@diABZI_lip_ groups being 8.3%, 13.0%, and 16.4%, respectively, underscoring the efficacy of DAC sequential STING agonist therapy (Figure 3B,C). The body weight curve of the mice is presented in Figure S1A (Supporting Information). This finding was consistent with the final tumor size and weight measurements, which were considerably lower in the sequential release group than in the monotherapy groups (Figure 3D,E).

To explore the impact of these treatments on the TME, immune cell subsets in the tumor and spleen were analyzed through flow cytometry (gating strategy detailed in Figure S1B, Supporting Information). The sequential release group exhibited augmented function of tumor‐infiltrating CD8+ T and NK cells, with higher TNF‐α and CD107a expression (Figure 3F–H). TNF‐α is a powerful pro‐inflammatory cytokine produced by various immune cells and a crucial player in regulating inflammation and tumor immunity.^[^ ^22^, ^23^ ^]^ CD107a is a marker of cytotoxic function in NK and T cells exposed during degranulation.^[^ ^24^ ^]^ By contrast, the monotherapy STING agonist group exhibited only slight or no changes in cytokine levels compared with the controls, thereby highlighting immune evasion within the TME of GBM cells with impaired STING pathways, which compromises immunotherapy efficacy. These results demonstrate that the SRHLS mitigates immunotherapy resistance in vivo, suggesting potential new therapeutic strategies for GBM.

### 2.4 SRHLS Effectively Inhibits Postsurgical Recurrence and Metastasis of GBM

Even after undergoing standard surgical resection and chemoradiotherapy, GBM patients encounter a formidable challenge with extremely high recurrence rates.^[^ ^25^ ^]^ In up to 80% of patients, recurrences occur within 2 cm of the initial tumor site, with the remaining appearing in distant brain regions, which indicates that invasive tumor cells had extended beyond the initial resection margins before treatment.^[^ ^26^ ^]^ Recurrent GBM are more aggressive, with patient survival being usually limited to within 12 months.^[^ ^27^ ^]^ Controlling GBM invasion and recurrence remains a key challenge in current treatments. A mouse GBM recurrence model was established with incomplete surgical resection (leaving ≈5% residual tumor) to test the SRHLS‐mediated persistent immune response against recurrence and metastasis. The schematic diagram and body weight curves of the mice are presented in Figure S2A,B (Supporting Information).

**Figure 4:**
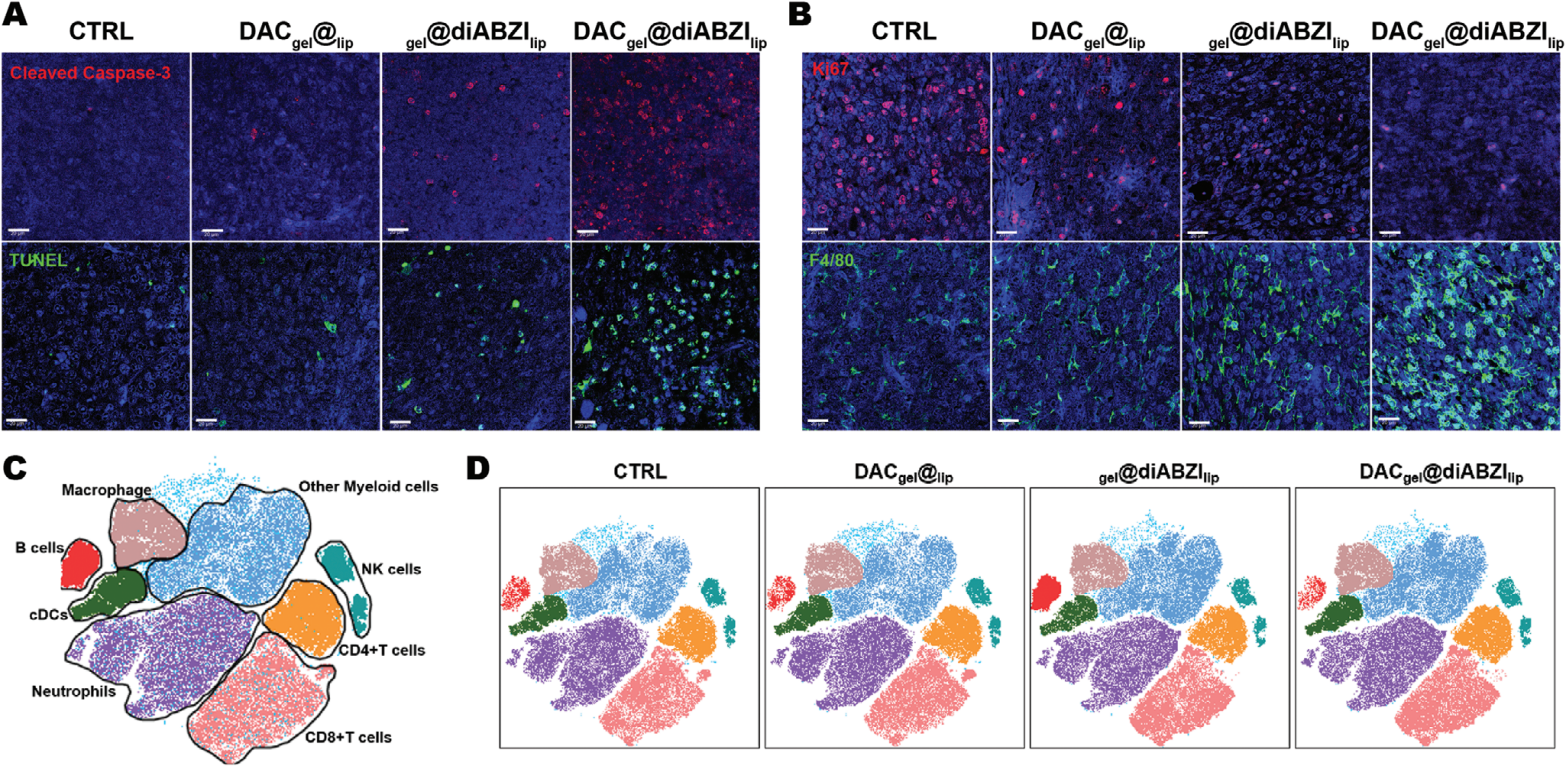
SRHLS substantially reduced post‐surgical recurrence and spread of GBM. A,B) Expression levels of cleaved caspase‐3 (red), Ki67 (red), TUNEL (green), and F4/80 (green) in tumor sections were assessed by immunofluorescence. Scale bar, 20µm. C,D) Dimensionality reduction and clustering analysis of flow cytometry data in recurrent tumors.

First, immunofluorescence analysis was performed on resected tumor tissues. Compared with the control and monotherapy groups, expression of the proliferation marker Ki‐67 was markedly downregulated, whereas that of the apoptosis markers TUNEL and cleaved‐caspase3 was upregulated by DAC_gel_@diABZI_lip_ treatment. This indicated that sequential therapy promoted tumor cell apoptosis and inhibited tumor proliferation. Additionally, tumor‐associated macrophages (TAMs) were markedly involved in the antitumor response, as evidenced by the increased expression of the macrophage marker F4/80 following DAC_gel_@diABZI_lip_ treatment (**Figure** **4**A,B). Because TAMs can be broadly classified into pro‐inflammatory, tumor‐suppressive phenotypes (M1‐like, CD86 positive) and anti‐inflammatory, tumor‐promoting phenotypes (M2‐like, CD206 positive),^[^ ^28^ ^]^ mRNA data of primary GBM patients from the CGGA database^[^ ^29^ ^]^ were analyzed. STING gene expression in tumor tissues was positively correlated with M1 macrophage surface markers such as CD80, CD86, and CD64 (Figure S3, Supporting Information). Future studies can further explore changes in relevant TAM subtypes.

After the tumor as surgically resected, the combination therapy group exhibited considerably reduced signs of recurrence according to tumor volume measurements, whereas the DAC_gel_@_lip_ and _gel_@diABZI_lip_ groups did not effectively suppress tumor regrowth (Figure S2C,E, Supporting Information). This suggested that the SRHLS‐generated antitumor immune response can sustain the inhibition of residual tumor cells after surgery. The flow cytometry analysis of recurrent tumors revealed no notable differences in the proportions of various infiltrating immune cells among the groups. This suggested that the functionality of immune cells was altered to maintain the continuous tumor‐killing activity (Figure 4C,D).

A metastasis model was further established by subcutaneously injecting tumor cells into the contralateral body side of the primary tumor. Similarly, DAC_gel_@diABZI_lip_ treatment inhibited metastatic tumor growth and progression compared with that in the other groups (Figure S2D,F, Supporting Information). These results demonstrate that the SRHLS generates persistent immune memory, thereby combating tumor recurrence and metastasis.

### 2.5 In Situ Tumor Therapy Demonstrates Strong Antitumor Immunity

**Figure 5:**
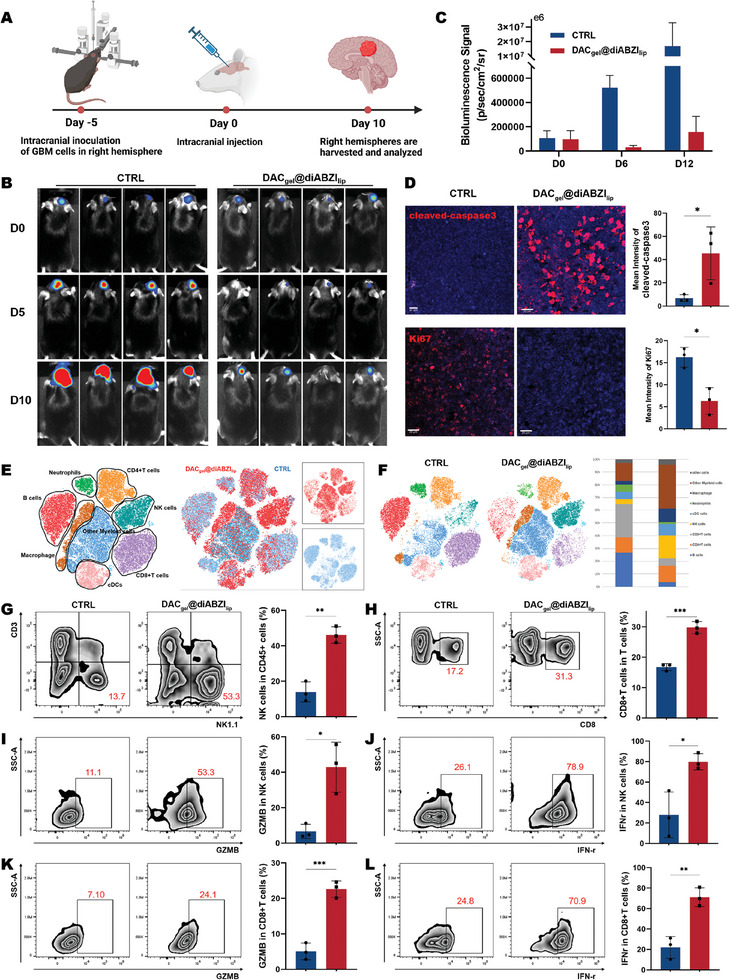
In situ SRHLS therapy elicited robust anti‐tumor immune responses. A) Schematic of the treatment protocol. B,C) In vivo luciferase fluorescence imaging before and after treatment with DAC_gel_@diABZI_lip_ (*n* = 4). D) Expression levels of cleaved caspase‐3 (red) and Ki67 (red) in tumor sections were assessed by immunofluorescence. Scale bar, 20µm. E,F) Dimensionality reduction and clustering analysis of flow cytometry data in tumors before and after treatment with DAC_gel_@diABZI_lip_. G) NK1.1 positive expression patterns in CD45+ cells (*n* = 3). H) CD8 positive expression patterns in CD3+ cells (*n* = 3). I–L) GZMB and IFN‐r positive expression patterns in NK and CD8+T cells (*n* = 3). ^*^ *p* < 0.05, ^**^ *p* < 0.01, ^***^ *p* < 0.001.

To further validate the antitumor efficacy and underlying action mechanisms of the SRHLS, we established an orthotopic GBM model to simulate clinical cases (**Figure** **5**A). In vivo fluorescence monitoring revealed that DAC_gel_@diABZI_lip_ greatly inhibited tumor growth by day 10 compared with the control group (saline) (Figure 5B,C). Immunofluorescence staining of coronal sections along the injection site revealed that DAC_gel_@diABZI_lip_ remarkably reduced tumor malignancy and promoted apoptosis, as indicated by Ki67 and cleaved‐caspase3 staining (Figure 5D). The body weight changes of the mice are shown in Figure S4 (Supporting Information).

Further dimensionality reduction and clustering analyses unveiled immune cell populations infiltrating the brain after drug delivery were substantially remodeled (Figure 5E,F). Monocyte‐macrophage and NK cell populations increased. The proportion of CD8+ T cells also increased among the total T cells (Figure 5G,H). NK cells have emerged as essential cytotoxic components in STING treatment.^[^ ^30^ ^]^ While the primary mechanism of tumor regression following STING treatment has been linked to the cytotoxic effects of CD8+ T cells,^[^ ^31^, ^32^ ^]^ recent data have also demonstrated the critical role of NK cells.^[^ ^33^ ^]^

On analyzing cytokine production by NK and CD8+ T cells, both cell types exhibited significant increases in the secretion of granzyme B (GZMB) and IFN‐γ (Figure 5I–L). GZMB is a hallmark molecule of cytotoxic cells and plays a central role in granzyme‐mediated target cell apoptosis.^[^ ^34^ ^]^ IFN‐γ, a downstream effector of STING pathway activation, has potent immunomodulatory functions and exerts broad‐spectrum antitumor effects.^[^ ^35^ ^]^ These results indicate that NK and CD8+ T cells synergistically contribute to orthotopic GBM regression.

### 2.6 SRHLS Reverses the Immunosuppressive Microenvironment

**Figure 6:**
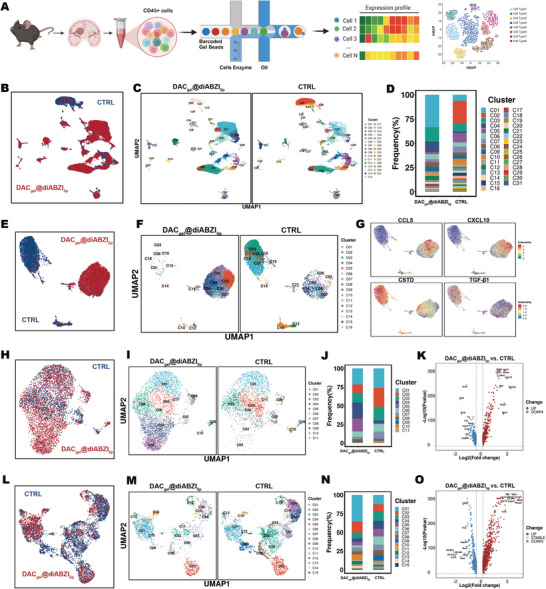
ScRNA‐seq analysis confirmed that the SRHLS promoted robust immune activation. A) Experimental design for scRNA‐seq analyses. B–D) Clustering and dimensional reduction analysis of immune cell classification in TME from control and treatment groups. E,F) UMAP dimensional reduction and re‐clustering analysis of macrophage populations. G) Expression distribution of M1 and M2 macrophage markers in macrophage clusters. H–K) UMAP dimensional reduction, re‐clustering analysis, and differential gene expression analysis of NK cells populations. L–O) UMAP dimensional reduction, re‐clustering analysis, and differential gene expression analysis of T cells populations.

To examine the detailed impact of the SRHLS on the GBM microenvironment, scRNA‐seq was conducted on GBM tissues from control and treatment groups (**Figure** **6**A). Each group comprised five samples of tumor‐bearing hemispheres, with CD45+ cells sorted through flow cytometry before sequencing. An analysis involving clustering and dimensional reduction techniques revealed notable shifts in cell types within the TME after treatment (Figure 6B–D). The overall proportion of macrophage populations (C01, C02, C16, C17, C21, and C23) remained stable, but distinct subtypes exhibited notable changes (cluster annotations are detailed in Table S1, Supporting Information). Additional UMAP dimensional reduction and re‐clustering highlighted that macrophage populations across treatment conditions were heterogeneous in nature (Figure 6E,F).

Specifically, macrophage populations before treatment demonstrated an elevation in CTSD and TGF‐β1 gene expression, indicative of an M2‐like pro‐tumor phenotype. By contrast, post‐treatment populations displayed increased CCL5 and CXCL10 expression, suggesting an M1‐like antitumor phenotype (Figure 6G). This illustrates SRHLS's capacity to shift TAMs toward an M1 phenotype.

Furthermore, the SRHLS treatment unveiled a notable rise in brain‐infiltrating NK cells (Figure 6H–J). Differential gene expression analysis identified upregulated genes after the treatment, including GZMB (a key cytotoxic effector molecule of NK and T cells), CXCL10 (important in inflammatory responses), and SPP1 (promoting NK cell survival and function) (Figure 6K). Similarly, T cells underwent functional reprogramming after the treatment, exhibiting high expression of GZMA (another critical cytotoxic effector molecule), GZMB, CXCL10, and CCL4 (involved in inflammation and immune responses) (Figure 6L–O). These findings indicate that the antitumor capabilities of NK and T cells increase following treatment. Additionally, we analyzed the expression of memory‐associated markers and found that markers such as ANXA1, CCL5, and GZMA were significantly elevated after combination therapy. This suggests that the upregulation of these memory markers may play a crucial role in the ability of the combinatorial treatment to effectively inhibit tumor recurrence and metastasis following surgical resection (Figure S5, Supporting Information).

In summary, the SRHLS remodels the TME toward an immune‐active phenotype by promoting the polarization of TAMs to an M1‐like phenotype and augmenting the antitumor functions of NK and T cells.

## 3 Conclusion

Our study presents compelling evidence that the SRHLS is a promising solution for enhancing the efficacy of immunotherapy for GBM. Based on the epigenetic reprogramming capabilities of DAC and the immune‐activating properties of STING agonists, the SRHLS facilitates a robust and sustained antitumor response. This dual‐phase release system addresses intrinsic STING signaling deficiencies in GBM cells and optimizes drug delivery to minimize side effects and costs. In vivo and in vitro results corroborate the system's potential to remodel the TME, thereby augmenting the cytotoxic functions of NK and CD8+ T cells. Future studies should focus on refining this delivery system and exploring its applicability in clinical settings, thus potentially transforming the therapeutic landscape for GBM and other recalcitrant malignancies.

## 4 Experimental Section

### Synthesis of OSA

To synthesize OSA, 20 g of sodium SA was dissolved in 2000 mL of deionized water. Subsequently, 10.8 g sodium periodic was added and stirred in the dark for 5 h, and then 30 mL glycol was added for another 1 h to terminate the reaction. The resultant solution was then placed in a dialysis bag and subjected to freeze‐drying for 48 h to obtain OSA.

### Synthesis of CEC

For the synthesis of CEC, 20 g CS was placed in 1000 mL deionized water, with 29.2 ml acrylic acid added, and stirred at 50 °C for 3 days. After the reaction, the pH of the solution was adjusted to 9–10 using sodium hydroxide. The solution was then placed in a dialysis bag for 48 h and subsequently freeze‐dried to yield CEC.

### Fabrication and Characterization of diABZI_lip_

DMPG (D130348) was procured from Shanghai Aladdin Biochemical Technology Co., Ltd. A total of 45 mg of DMPG was dissolved in 3 mL of chloroform. The solution was transferred to a single‐neck round‐bottom flask and evaporated for 1 h to remove the chloroform. Following this, 4 mL of PBS buffer containing a specified amount of diABZI was added, and rotary evaporation was continued to disperse the diABZI_lip_. The morphology of the liposomes was examined using SEM, while the size and polydispersity index (PDI) were determined via dynamic light scattering.

### Fabrication and Morphology of DAC_gel_@diABZI_lip_

Solution A was prepared by dissolving 0.5 g of OSA in 5 mL of the previously obtained diABZI_lip_ solution. Solution B was prepared by dissolving 0.1 g of CEC in deionized water. Equal volumes of Solutions A and B were uniformly mixed using a double injection phase method, resulting in the formation of DAC_gel_@diABZI_lip_ after ≈15 s. The hydrogel network structure and the distribution of liposomes within the hydrogel were observed using SEM.

### Release Assay of DAC_gel_@diABZI_lip_

The final concentration of the two drugs is 0.5mg mL^−1^ for DAC and 2mg mL^−1^ for diABZI. To evaluate the release of DAC_gel_@diABZI_lip_ in vitro, 1 mL of the gel was immersed in 50 mL of PBS in a centrifuge tube and placed on a shaking table at 37 °C. At fixed time points, 100 µL of the supernatant was withdrawn and replaced with an equal volume of PBS to maintain a constant volume. Since DAC and diABZI cannot be accurately distinguished in the UV spectrum, they were separated using gel chromatography. The concentrations and release rates of DAC and diABZI at each time point were calculated based on elution time and peak area. In vivo release tests were conducted on mice, with fluorescence intensity and localization monitored using an in vivo imaging system.

### Cell Culture

The human glioblastoma cell line LN229 and murine glioblastoma cell lines GL261 and G422 were purchased from Pricella Life Science & Technology Co., Ltd (Wuhan, China) and Cellverse Bioscience Technology Co., Ltd (Shanghai, China). GBM patient‐derived stem cells (GSCs) GBM#020 and GBM#021 were generously gifted by the Department of Neurosurgery, Second Affiliated Hospital, School of Medicine, Zhejiang University.^[^ ^36^ ^]^ GBM#020 and GBM#021 were cultured in DMEM/F‐12 medium (#11 320 033, Thermo Fisher Scientific, USA) supplemented with 1% B27 (Thermo Fisher Scientific #17 504 044), 1% GlutaMAX (Thermo Fisher Scientific #35 050 061), EGF (20 ng mL^−1^; Novoprotein Scientific; Suzhou, China), and FGF (20 ng mL^−1^; Novoprotein Scientific). The murine primary glioblastoma cell line QPP12 was cultured in NeuroCult NSC Basal Medium (mouse) supplemented with 10% NeuroCult NSC Proliferation Supplement (STEMCELL Technologies, Vancouver, Canada), EGF (20 ng mL^−1^), and FGF (20 ng mL^−1^; both from Novoprotein Scientific). LN229, GL261, and G422 cells were cultured in DMEM medium (Thermo Fisher Scientific #11 965 092) supplemented with 10% fetal bovine serum (FBS; Thermo Fisher Scientific #A5669701).

### In Vitro DAC and STING Agonist Treatment

Human and mouse GBM cell lines (3×10^5^ cells wel^−1^l in 6‐well plates) were treated with 100–250 nm DAC (MCE #HY‐A0004) dissolved in culture medium and replaced daily for 5 days, followed by addition of 10 µg mL^−1^ 2′3“‐cGAMP (MCE #HY‐100564). Protein was harvested at 24 h post 2′3”‐cGAMP treatment.

### ELISA

Cytokine secretion levels in the cell culture supernatants were measured through ELISA. Commercial ELISA kits were used according to the manufacturers' protocols: human IFN‐β (Human IFN‐β ELISA Kit, Cusabio, Cat# CSB‐E09889 h) and human CXCL10 (Human IP‐10 ELISA Kit, Raybiotech, Cat# P02778).

### Western Blot

The cells were lysed with RIPA buffer (Thermo Fisher Scientific #89 900) supplemented with a protease inhibitor cocktail (MCE #HY‐K0010). Total cell lysates were quantified for protein content determination (Beyotime) and subjected to immunoblotting by using specific antibodies: DNMT1 (Cell Signaling Technology #5032T), DNMT3A (Proteintech #20954‐1‐AP), STING (Cell Signaling Technology #13647S), and β‐tubulin (Cell Signaling Technology #2146S).

### Pyrosequencing

Using the QIAamp Fast DNA Tissue Kit (QIAGEN # 51 404, Germany) to extract Genomic DNA. Bisulfite conversion was performed using the Qiagen EpiTect Bisulfite Kit (QIAGEN #59 104). Primers were designed and PCR amplification was performed. Based on the sequence design information calculated using pyrosequencing software, a substrate mixture, an enzyme mixture, and four types of dNTPs were sequentially added to the reagent chamber. The reagent chamber and 96‐well reaction plate were placed in the pyrosequencing instrument (QIAGEN) for the reaction.

### In Vitro Flow Cytometry

Adhesive cells were digested with trypsin (Thermo Fisher Scientific #25 200 114) to form a single‐cell suspension, whereas the suspension cells were digested with TrypLE Select (Thermo Fisher Scientific #A1217701). After blocking was performed, the cells were stained on ice. APC anti‐human HLA‐A, B, C (#311 410) and APC anti‐mouse H‐2Kd (#116 620) were purchased from BioLegend. Flow cytometry analysis was performed using the CytoFLEX LX Series Flow Cytometer (Beckman Coulter, USA). The results were analyzed using FlowJo (TreeStar, Ashland, OR, USA).

### Establishment of the GBM Model

Female C57BL/6 mice (age: 6–8 weeks) were obtained from the Zhejiang University Medical Center in Hangzhou, China. Animal experiments were conducted according to the Chinese Animal Protection Law and have been approved by the First Affiliated Hospital, Zhejiang University School of Medicine (Approval number: 2024‐1214). Efforts were taken to minimize or avoid animal suffering so as to improve their welfare. To produce the subcutaneous GBM model, the C57BL/6 mice were subcutaneously inoculated with 3 × 10^5^ cancer cells. To establish the orthotopic GBM model, 2 × 10^5^ cancer cells were inoculated into the right cerebral hemisphere of the C57BL/6 mice by using a stereotactic apparatus and a microinjection pump.

### Tumor Immune Microenvironment Assay Using Flow Cytometry

Tissues were cut into uniform fragments and placed in 4 mL RPMI‐1640 medium (Gibco #11 875 093) containing 10% FBS, collagenase IV (Absin # abs47048003), DNase (MCE #HY‐108882), and hyaluronidase (Sigma #H4272). The tubes were incubated at 37 °C on a shaker at 145 rpm for 60 min. After the tissues were digested, the mixture was filtered to obtain a cell suspension. The cells were resuspended in 36% Percoll solution (Cytiva #17089109‐1), and immune cells were collected through density gradient centrifugation. The cells were stimulated with a leukocyte activation cocktail (BD Pharmingen # 550 583) for 4–6 h. Cells were blocked with purified rat anti‐mouse CD16/CD32 (BD Pharmingen # 553 141), and stained for cell surface markers. The cells were fixed and permeabilized using the Fixation/Permeabilization Kit (BD Pharmingen # 554 714) and stained for intracellular epitopes. PE‐CY7 GZMB monoclonal antibody (#25‐8898‐82) was purchased from Thermo Fisher Scientific. Fixable viability stain 510 (#564 406), APC‐Cy7 rat anti‐mouse CD45 (#557 659), FITC hamster anti‐mouse CD3e (#553 061), BUV395 rat anti‐mouse CD8a (#563 786), BV421 mouse anti‐mouse NK‐1.1 (#562 921), PE rat anti‐mouse CD107a (#558 661), BB700 rat anti‐mouse TNF (#566 510), Alexa Fluor 647 rat anti‐mouse IFN‐γ (#557 735), PerCP‐Cy5.5 rat anti‐mouse Ly‐6G (#560 602), and PE‐Cy7 hamster anti‐mouse CD11c (#561 022) were all purchased from BD Pharmingen. Brilliant Violet 785 anti‐mouse/human CD11b (#101 243), PE/Dazzle 594 anti‐mouse F4/80 (#123 145), PE anti‐mouse CD19 (#152 407), and Alexa Fluor 700 anti‐mouse I‐A/I‐E (#107 621) were all purchased from BioLegend. Flow cytometry was performed using an LSRFortessa Cell Analyzer (BD Biosciences, USA) and Cytek Aurora Full Spectrum Flow Cytometer (Cytek Biosciences, USA). The results were analyzed using FlowJo (TreeStar, USA).

### Immunofluorescence

Tumor or brain tissues were fixed in formalin solution, embedded in paraffin, and sectioned. The sections were baked at 60–70 °C for 30 min, deparaffinized in xylene, and rehydrated in graded ethanol solutions. Antigen retrieval was performed in citrate buffer (Sigma #C9999) at 98 °C for 20 min, and the sections were cooled to room temperature. The sections were sequentially washed in 1× PBS, PBST, TBS, and TBST solutions. The tissues were first incubated with primary antibodies Ki67 (Abcam #ab16667), F4/80 (Abcam #ab300421), and cleaved‐caspase3 (CST #9661T) overnight at 4 °C, and then with secondary antibodies at room temperature for 60 min. DAPI staining and mounting were performed using Antifade Mountant (Thermo Fisher Scientific #P36935). Using the TUNEL Apoptosis Assay Kit (Beyotime #C1090) to perform TUNEL staining. Images were captured using a STELLARIS 8 confocal microscope (Leica Microsystems, Germany).

### Single‐Cell RNA Sequencing

scRNA‐seq was performed using the 10x Genomics platform (version 3ʹ Kit v3.1). Tumor‐bearing cerebral hemispheres were enzymatically digested as described above. Lymphocytes were enriched through a percoll gradient. The cells were stained with the live/dead dye and CD45 antibody. Using a cell sorter, live CD45+ cells were sorted into RPMI‐1640 medium. For scRNA‐Seq, the samples were processed and libraries were constructed according to the manufacturer's guidelines. Data were analyzed using the Seurat (version 4.3.0).^[^ ^37^ ^]^

### Statistical Analysis

Data were analyzed using GraphPad Prism 9 (GraphPad Inc., La Jolla, CA, USA). One‐way ANOVA and Student's t‐test performed to determine between‐group differences. *p* < 0.05 was considered statistically significant (^*^ *p* < 0.05, ^**^ *p* < 0.01, and ^***^ *p* < 0.001).

## Conflict of Interest

The authors declare no conflict of interest.

## Author Contributions

H.Y. and W.L. contributed equally to this work and shared co‐first authorship. H.Y. and W.L. conceived and designed the project. H.Y., W.L., K.D., and J.W. performed experiments. H.Y., W.L., and S.W. analyzed the data. H.Y. and W.L. wrote the manuscript. C.W., L.W., Q.T., X.Y., K.J., and D.Y. provided reagents and materials. X.W., S.C., and S.Y. conceived, designed, and supervised the project. All authors read and approved the final version of manuscript.

## Supporting information



Supporting Information

## Data Availability

The data that support the findings of this study are available from the corresponding author upon reasonable request.

## References

[1] L. Rong , N. Li , Z. Zhang , J. Exp. Clin. Cancer. Res. 2022, 41, 142. 35428347 10.1186/s13046-022-02349-7 PMC9013078

[2] A. C. Tan , D. M. Ashley , G. Y. Lopez , M. Malinzak , H. S. Friedman , M. Khasraw , CA Cancer. J. Clin. 2020, 70, 299. 32478924 10.3322/caac.21613

[3] J. Ji , K. Ding , B. Cheng , X. Zhang , T. Luo , B. Huang , H. Yu , Y. Chen , X. Xu , H. Lin , J. Zhou , T. Wang , M. Jin , A. Liu , D. Yan , F. Liu , C. Wang , J. Chen , F. Yan , L. Wang , J. Zhang , S. Yan , J. Wang , X. Li , G. Chen , Adv. Sci. 2024, 11, e2304609. 10.1002/advs.202304609 PMC11022718 38342629

[4] L. R. Schaff , I. K. Mellinghoff , JAMA, J. Am. Med. Assoc. 2023, 329, 574. 10.1001/jama.2023.0023 PMC11445779 36809318

[5] M. E. Hegi , A. C. Diserens , T. Gorlia , M. F. Hamou , N. de Tribolet , M. Weller , J. M. Kros , J. A. Hainfellner , W. Mason , L. Mariani , J. E. Bromberg , P. Hau , R. O. Mirimanoff , J. G. Cairncross , R. C. Janzer , R. Stupp , N. Engl. J. Med. 2005, 352, 997. 15758010 10.1056/NEJMoa043331

[6] D. A. Reardon , A. A. Brandes , A. Omuro , P. Mulholland , M. Lim , A. Wick , J. Baehring , M. S. Ahluwalia , P. Roth , O. Bahr , S. Phuphanich , J. M. Sepulveda , P. De Souza , S. Sahebjam , M. Carleton , K. Tatsuoka , C. Taitt , R. Zwirtes , J. Sampson , M. Weller , JAMA Oncol. 2020, 6, 1003. 32437507 10.1001/jamaoncol.2020.1024 PMC7243167

[7] A. Omuro , A. A. Brandes , A. F. Carpentier , A. Idbaih , D. A. Reardon , T. Cloughesy , A. Sumrall , J. Baehring , M. van den Bent , O. Bahr , G. Lombardi , P. Mulholland , G. Tabatabai , U. Lassen , J. M. Sepulveda , M. Khasraw , E. Vauleon , Y. Muragaki , A. M. Di Giacomo , N. Butowski , P. Roth , X. Qian , A. Z. Fu , Y. Liu , V. Potter , A. G. Chalamandaris , K. Tatsuoka , M. Lim , M. Weller , Neuro Oncol. 2023, 25, 123. 35419607 10.1093/neuonc/noac099 PMC9825306

[8] H. Konno , S. Yamauchi , A. Berglund , R. M. Putney , J. J. Mule , G. N. Barber , Oncogene 2018, 37, 2037. 29367762 10.1038/s41388-017-0120-0 PMC6029885

[9] L. Gao , Y. Zhang , S. Wang , P. Kong , Y. Su , J. Hu , M. Jiang , H. Bai , T. Lang , J. Wang , L. Liu , T. Yang , X. Huang , F. Liu , S. Lou , Y. Liu , C. Zhang , H. Liu , L. Gao , J. Liu , L. Zhu , Q. Wen , T. Chen , P. Wang , J. Rao , M. Mao , C. Wang , X. Duan , L. Luo , X. Peng , et al., J. Clin. Oncol. 2020, 38, 4249. 33108244 10.1200/JCO.19.03277 PMC7768335

[10] R. Falahat , A. Berglund , R. M. Putney , P. Perez‐Villarroel , S. Aoyama , S. Pilon‐Thomas , G. N. Barber , J. J. Mule , Proc. Natl. Acad. Sci. USA 2021, 118, e2013598118. 33827917 10.1073/pnas.2013598118 PMC8053941

[11] R. Falahat , A. Berglund , P. Perez‐Villarroel , R. M. Putney , I. Hamaidi , S. Kim , S. Pilon‐Thomas , G. N. Barber , J. J. Mule , Nat. Commun. 2023, 14, 1573. 36949064 10.1038/s41467-023-37217-1 PMC10033671

[12] Y. Xiao , Y. Gu , L. Qin , L. Chen , X. Chen , W. Cui , F. Li , N. Xiang , X. He , Colloids. Surf. B Biointerfaces. 2021, 200, 111581. 33524696 10.1016/j.colsurfb.2021.111581

[13] L. E. Kass , J. Nguyen , Wiley. Interdiscip. Rev. Nanomed. Nanobiotechnol. 2022, 14, e1756. 34532989 10.1002/wnan.1756 PMC9811486

[14] C. Tan , J. Wang , B. Sun , Biotechnol. Adv. 2021, 48, 107727. 33677025 10.1016/j.biotechadv.2021.107727

[15] M. Hamidi , A. Azadi , P. Rafiei , Adv. Drug Delivery Rev. 2008, 60, 1638. 10.1016/j.addr.2008.08.002 18840488

[16] A. J. Clasky , J. D. Watchorn , P. Z. Chen , F. X. Gu , Acta Biomater. 2021, 122, 1. 33352300 10.1016/j.actbio.2020.12.030

[17] L. Zhao , Z. Feng , Y. Lyu , J. Yang , L. Lin , H. Bai , Y. Li , Y. Feng , Y. Chen , Int. J. Biol. Macromol. 2023, 230, 123231. 36641017 10.1016/j.ijbiomac.2023.123231

[18] N. Bhattarai , J. Gunn , M. Zhang , Adv Drug Delivery Rev 2010, 62, 83. 10.1016/j.addr.2009.07.019 19799949

[19] M. Liu , S. Guo , J. M. Hibbert , V. Jain , N. Singh , N. O. Wilson , J. K. Stiles , Cytokine Growth Factor Rev. 2011, 22, 121. 21802343 10.1016/j.cytogfr.2011.06.001 PMC3203691

[20] L. B. Ivashkiv , L. T. Donlin , Nat. Rev. Immunol. 2014, 14, 36. 24362405 10.1038/nri3581 PMC4084561

[21] D. Chowell , L. Morris , C. M. Grigg , J. K. Weber , R. M. Samstein , V. Makarov , F. Kuo , S. M. Kendall , D. Requena , N. Riaz , B. Greenbaum , J. Carroll , E. Garon , D. M. Hyman , A. Zehir , D. Solit , M. Berger , R. Zhou , N. A. Rizvi , T. A. Chan , Science 2018, 359, 582. 29217585 10.1126/science.aao4572 PMC6057471

[22] B. B. Aggarwal , Nat. Rev. Immunol. 2003, 3, 745. 12949498 10.1038/nri1184

[23] T. Horiuchi , H. Mitoma , S. Harashima , H. Tsukamoto , T. Shimoda , Rheumatology 2010, 49, 1215. 20194223 10.1093/rheumatology/keq031 PMC2886310

[24] E. L. Eskelinen , Y. Tanaka , P. Saftig , Trends Cell Biol. 2003, 13, 137. 12628346 10.1016/s0962-8924(03)00005-9

[25] B. Campos , L. R. Olsen , T. Urup , H. S. Poulsen , Oncogene 2016, 35, 5819. 27041580 10.1038/onc.2016.85

[26] A. A. Brandes , A. Tosoni , E. Franceschi , G. Sotti , G. Frezza , P. Amista , L. Morandi , F. Spagnolli , M. Ermani , J. Clin. Oncol. 2009, 27, 1275. 19188675 10.1200/JCO.2008.19.4969

[27] W. Taal , H. M. Oosterkamp , A. M. Walenkamp , H. J. Dubbink , L. V. Beerepoot , M. C. Hanse , J. Buter , A. H. Honkoop , D. Boerman , F. Y. de Vos , W. N. Dinjens , R. H. Enting , M. J. Taphoorn , F. W. van den Berkmortel , R. L. Jansen , D. Brandsma , J. E. Bromberg , I. van Heuvel , R. M. Vernhout , B. van der Holt , M. J. van den Bent , Lancet Oncol. 2014, 15, 943. 25035291 10.1016/S1470-2045(14)70314-6

[28] I. Vitale , G. Manic , L. M. Coussens , G. Kroemer , L. Galluzzi , Cell Metab. 2019, 30, 36. 31269428 10.1016/j.cmet.2019.06.001

[29] Z. Zhao , K. N. Zhang , Q. Wang , G. Li , F. Zeng , Y. Zhang , F. Wu , R. Chai , Z. Wang , C. Zhang , W. Zhang , Z. Bao , T. Jiang , Genomics Proteomics Bioinformatics 2021, 19, 1. 33662628 10.1016/j.gpb.2020.10.005 PMC8498921

[30] C. J. Nicolai , N. Wolf , I. C. Chang , G. Kirn , A. Marcus , C. O. Ndubaku , S. M. Mcwhirter , D. H. Raulet , Sci. Immunol. 2020, 5, eaaz2738. 32198222 10.1126/sciimmunol.aaz2738 PMC7228660

[31] O. Demaria , A. De Gassart , S. Coso , N. Gestermann , J. Di Domizio , L. Flatz , O. Gaide , O. Michielin , P. Hwu , T. V. Petrova , F. Martinon , R. L. Modlin , D. E. Speiser , M. Gilliet , Proc. Natl. Acad. Sci. USA 2015, 112, 15408. 26607445 10.1073/pnas.1512832112 PMC4687570

[32] E. Curran , X. Chen , L. Corrales , D. E. Kline , T. J. Dubensky , P. Duttagupta , M. Kortylewski , J. Kline , Cell Rep. 2016, 15, 2357. 27264175 10.1016/j.celrep.2016.05.023 PMC5116809

[33] A. Marcus , A. J. Mao , M. Lensink‐Vasan , L. Wang , R. E. Vance , D. H. Raulet , Immunity 2018, 49, 754. 30332631 10.1016/j.immuni.2018.09.016 PMC6488306

[34] A. Aubert , K. Jung , S. Hiroyasu , J. Pardo , D. J. Granville , Nat. Rev. Rheumatol 2024, 20, 361. 38689140 10.1038/s41584-024-01109-5

[35] Q. Chen , L. Sun , Z. J. Chen , Nat. Immunol. 2016, 17, 1142. 27648547 10.1038/ni.3558

[36] Y. Chen , X. Xu , K. Ding , T. Tang , F. Cai , H. Zhang , Z. Chen , Y. Qi , Z. Fu , G. Zhu , Z. Dou , J. Xu , G. Chen , Q. Wu , J. Ji , J. Zhang , J. Exp. Clin. Cancer. Res. 2024, 43, 39. 38303029 10.1186/s13046-024-02964-6 PMC10835844

[37] T. Stuart , A. Butler , P. Hoffman , C. Hafemeister , E. Papalexi , W. R. Mauck , Y. Hao , M. Stoeckius , P. Smibert , R. Satija , Cell 2019, 177, 1888. 31178118 10.1016/j.cell.2019.05.031 PMC6687398

