# Supplementary material for: Sequential Release HydroLipo System for STING Gene Epigenetic Reprogramming and Immune Activation in Glioblastoma
Source: Adv Sci (Weinh). 2024 Dec 11;12(5):2408323. doi: 10.1002/advs.202408323 (PMC11792002; doi:10.1002/advs.202408323)
Supplement: Supplementary file 1 — Supporting Information [file ADVS-12-2408323-s001.docx]

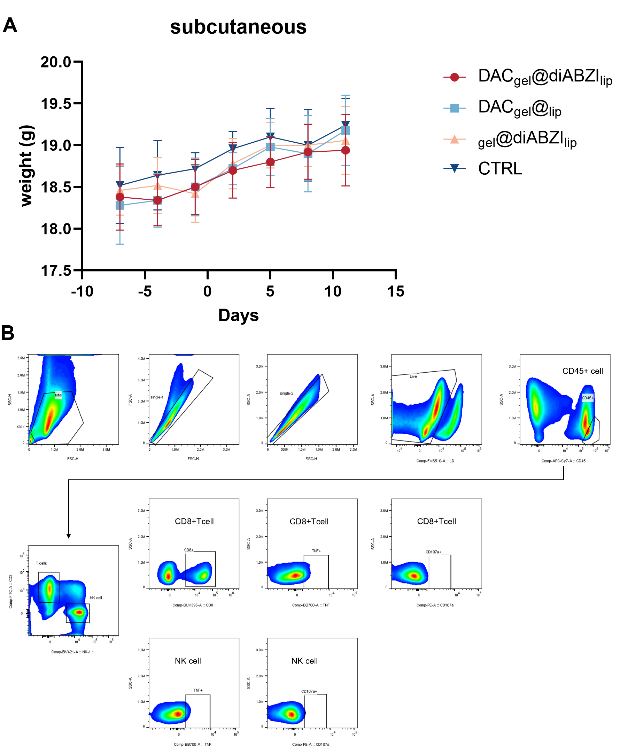


Supplementary Figure S1. A) Body weight curve of subcutaneous tumor bearing mice (n=5). B) Gating strategy for NK and CD8+ T cells in C57BL/6 mice tumors.


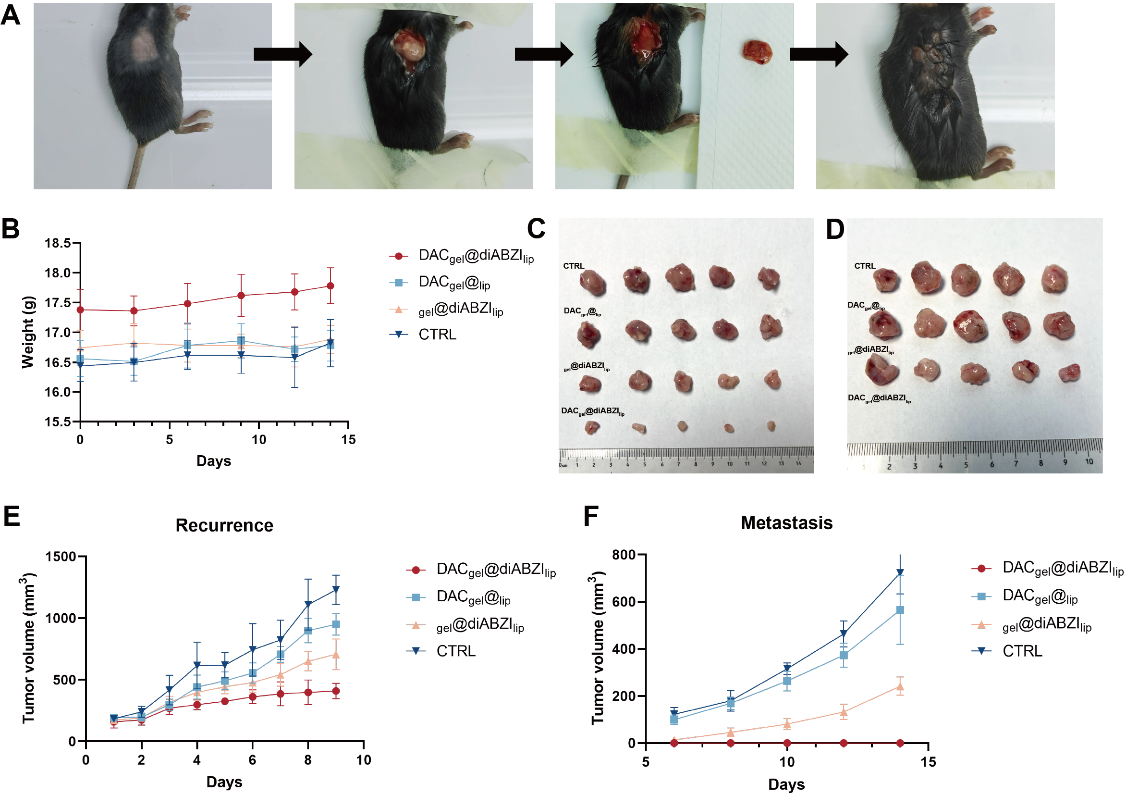


Supplementary Figure S2. A) Surgery was conducted on C57BL/6 mice in a sterile environment to excise the majority of the tumor tissue, leaving approximately 5% of the tumor to continue growing. After suturing, the site was thoroughly disinfected. B) Body weight curve of tumor bearing mice (n=5). C-D) Bright field image of excised tumor tissue on the recurrence side (n=5) and the metastasis side (n=5). DAC_gel_@diABZI_lip_ treatment markedly inhibited recurrent tumor (E) and metastatic tumor (F) growth according to tumor volume measurements (n=5).


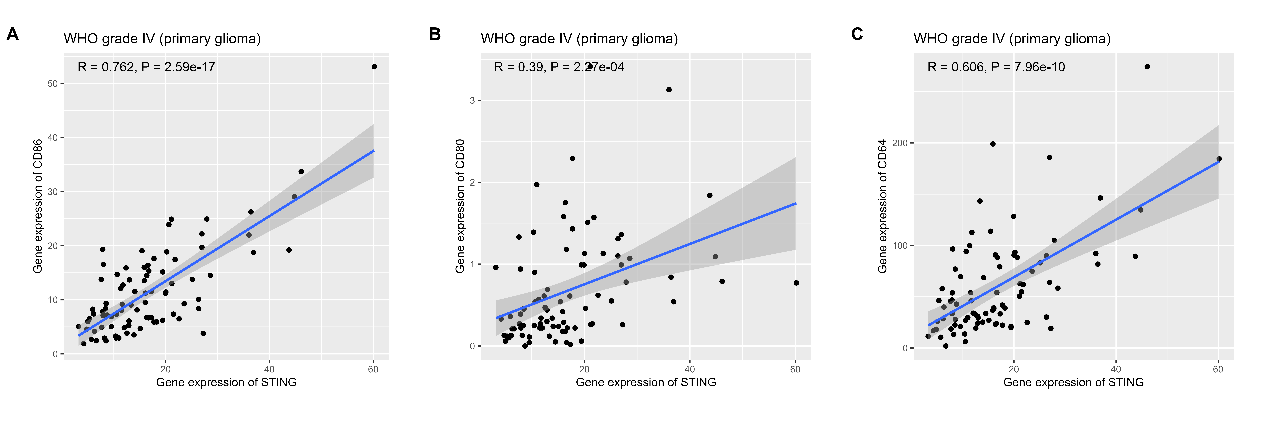


Supplementary Figure S3. STING gene expression in tumor tissues was positively correlated with M1 macrophage surface markers such as CD80, CD86, and CD64 in CGGA database (<http://www.cgga.org.cn>).


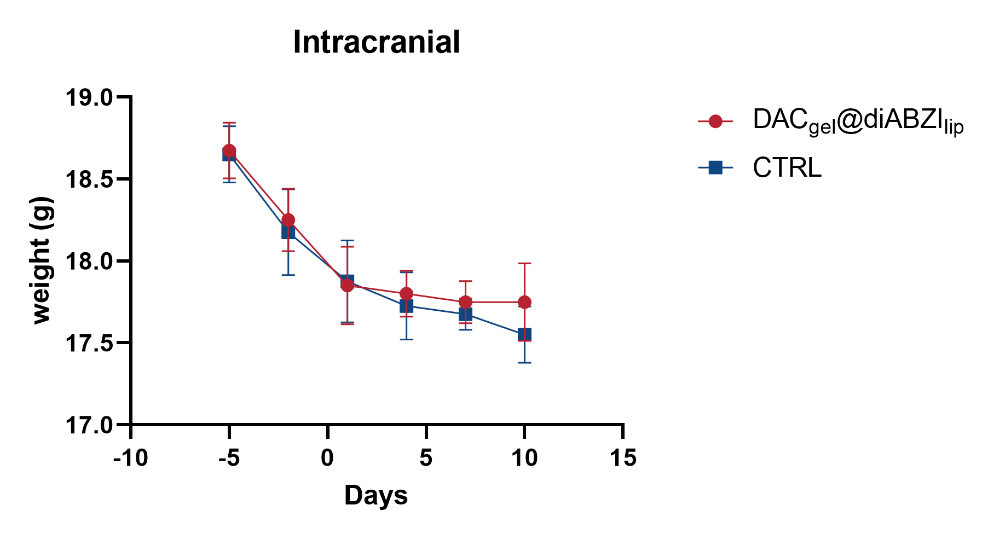


Supplementary Figure S4. Body weight curve of intracranial tumor bearing mice (n=4).


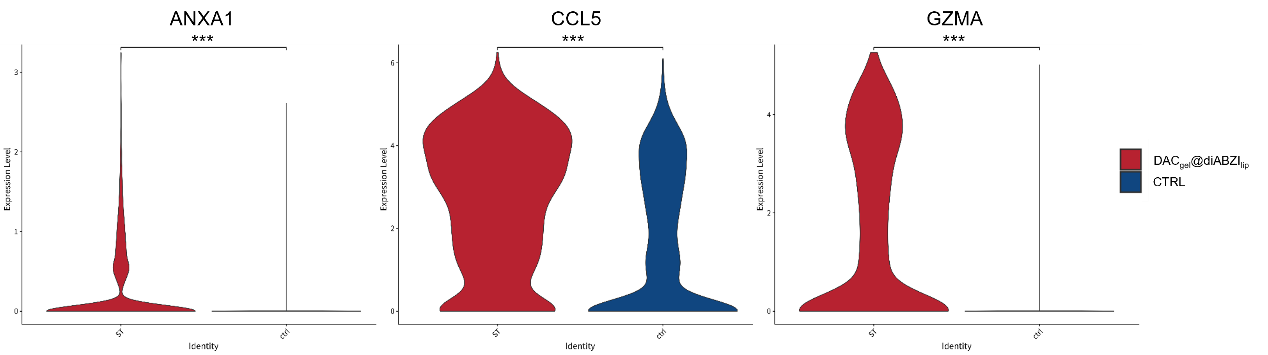


Supplementary Figure S5. Expression of memory-associated markers following combinatorial treatment. *p < 0.05, **p < 0.01, and ***p < 0.001.

| Subgroup | Define | Marker gene |
| --- | --- | --- |
| C01 | Macrophage | Ctss, Apoe, Mafb, Cfb |
| C02 | Macrophage(Microglia) | Cx3cr1, C1qa, C1qb, C1qc, Mertk |
| C03 | NK | Gzma, Gzmb, Nkg7, Prf1 |
| C04 | Monocytes | Ly6c2, Cxcl10, Ifit3, F13a1, Fn1 |
| C05 | T cell | CD3d, CD3e, CD3g, Trac, Trbc2 |
| C06 | cDC2 | Cd209a, H2-DMb1, Napsa |
| C07 | B cell | CD79a, CD79b, Ms4a1 |
| C08 | NK | Gzma, Gzmb, Nkg7, Prf1 |
| C09 | T cell | Trac, Trbc2, CD28 |
| C10 | T cell | CD3d, CD3e, CD3g, Trac, Trbc2 |
| C11 | cDC1 | Ccl22, Fscn1, Ccr7, Socs2 |
| C12 | Neutrophils | S100a8, S100a9, Clec4d |
| C13 | Cycling T cell | Top2a, Mki67, Trac, Trbc2, CD3g |
| C14 | pDC | Siglech, Tcf4, Runx2, Cox6a2 |
| C15 | Non-immune cell | Ttr, Enpp2 |
| C16 | Macrophage(Microglia) | Cx3cr1, C1qa, C1qb, C1qc, Mertk |
| C17 | Macrophage-like | Mafb, Adgre1 |
| C18 | γδT | CD3g, Trdc |
| C19 | cDC1 | Clec9a, Wdfy4, Xcr1 |
| C20 | Monocytes | Itgal, Adgre4, Ear2, Nr4a1 |
| C21 | Macrophage(Microglia) | Cx3cr1, C1qa, C1qb, C1qc, Aif1 |
| C22 | T cell-like | Skap1, Tox, Itk, Trbc1 |
| C23 | Macrophage(Microglia) | Cx3cr1, C1qa, C1qb, C1qc, Mertk |
| C24 | Non-immune cell | Cldn5, Ly6a, Ly6e(Endothelial) |
| C25 | Non-immune cell | Igfbp7, Col3a1, Dcn(Fibroblast) |
| C26 | B cell | CD79a, CD79b, Ms4a1 |
| C27 | Non-immune cell | Distribution disorder |
| C28 | cDC1 | Ccl22, Fscn1, Serpinb6b, Cacnb3 |
| C29 | meaningless | meaningless |
| C30 | cDC1 | Ccl22, Fscn1, Serpinb6b, Ccr7 |
| C31 | Non-immune cell | Plp1, Apod (oligodendrocytes) |

Table S1. Subgroup definition for scRNA-seq.
